# Supplementary material for: Complex‐centric proteome profiling by SEC‐SWATH‐MS
Source: Mol Syst Biol. 2019 Jan 14;15(1):e8438. doi: 10.15252/msb.20188438 (PMC6346213; doi:10.15252/msb.20188438)
Supplement: Supplementary file 8 — Dataset EV7 [file MSB-15-e8438-s008.zip › feature_plots_string/Q96RL1.pdf]

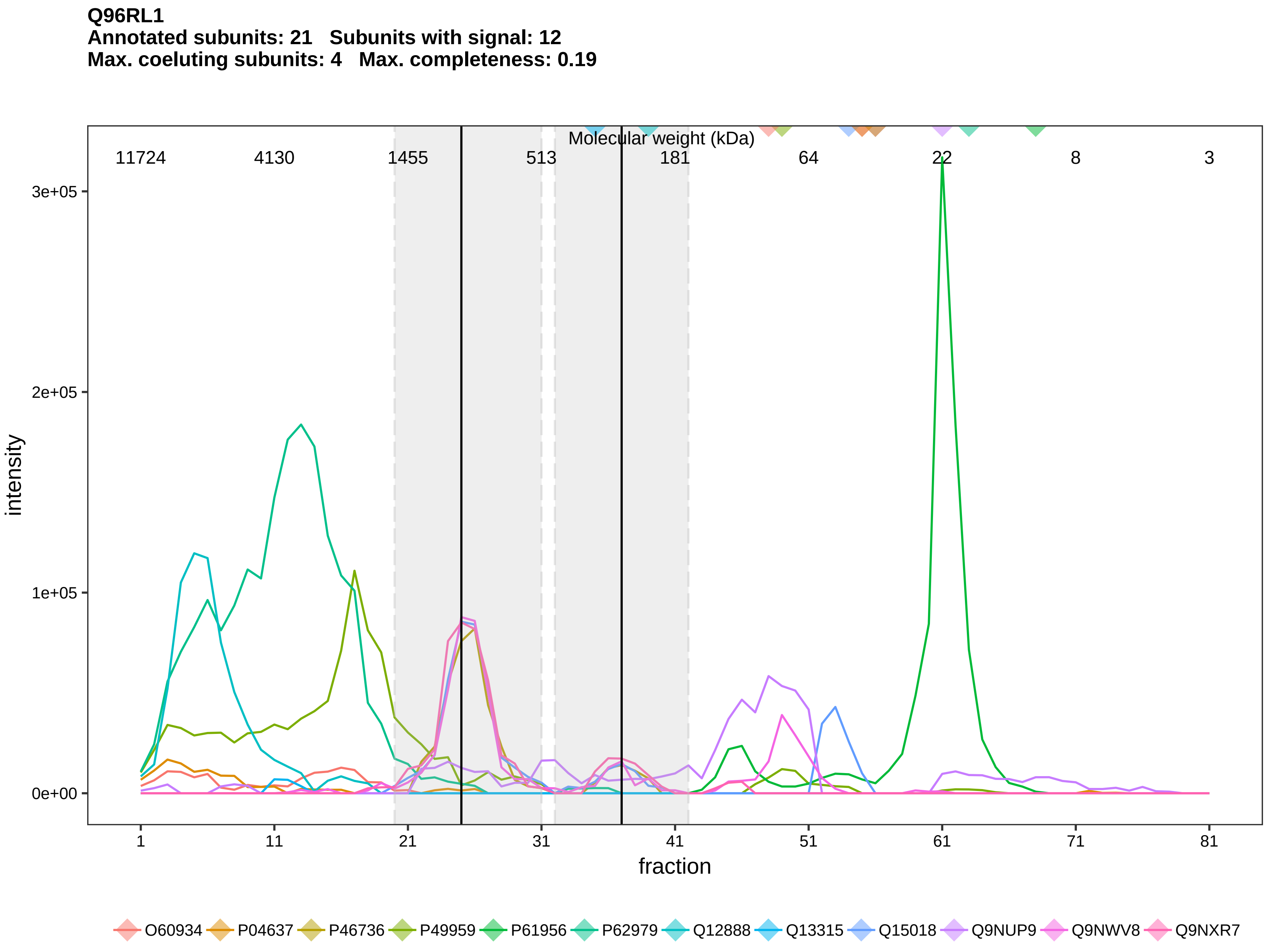
Q96RL1
Annotated subunits: 21 Subunits with signal: 12
Max. coeluting subunits: 4 Max. completeness: 0.19
Molecular weight (kDa)
11724
4130
1455
513
181
64
22
8
3
intensity
3e+05
2e+05
1e+05
0e+00
1
11
21
31
41
51
61
71
81
fraction
O60934
P04637
P46736
P49959
P61956
P62979
Q12888
Q13315
Q15018
Q9NUP9
Q9NWV8
Q9NXR7
